# Validation of aortic valve 4D flow analysis and myocardial deformation by cardiovascular magnetic resonance in patients after the arterial switch operation

**DOI:** 10.1186/s12968-019-0527-6

**Published:** 2019-03-18

**Authors:** W. H. S. van Wijk, J. M. P. J. Breur, J. J. M. Westenberg, M. M. P. Driessen, F. J. Meijboom, B. Driesen, E. C. de Baat, P. A. F. M. Doevendans, T. Leiner, H. B. Grotenhuis

**Affiliations:** 1 Department of Pediatric Cardiology, University Medical Center Utrecht / Wilhelmina Children’s Hospital, Utrecht, The Netherlands; 2 Department of Radiology, Leiden University Medical Center, Leiden, The Netherlands; 3 Department of Cardiology, University Medical Center Utrecht, Utrecht, The Netherlands; 4 Department of Cardiology, Radboud UMC Nijmegen, Nijmegen, The Netherlands; 5 Netherlands Hearth Institute, Utrecht, The Netherlands; 6 Central Military Hospital, Utrecht, The Netherlands; 7 Department of Radiology, University Medical Center Utrecht, Postal box 85090, 3508 AB Utrecht, The Netherlands

**Keywords:** CMR, 4D flow, Feature tracking, TGA, Arterial switch operation

## Abstract

**Background:**

Aortic regurgitation (AR) and subclinical left ventricular (LV) dysfunction expressed by myocardial deformation imaging are common in patients with transposition of the great arteries after the arterial switch operation (ASO). Echocardiographic evaluation is often hampered by reduced acoustic window settings. Cardiovascular magnetic resonance (CMR) imaging provides a robust alternative as it allows for comprehensive assessment of degree of AR and LV function. The purpose of this study is to validate CMR based 4-dimensional flow quantification (4D flow) for degree of AR and feature tracking strain measurements for LV deformation assessment in ASO patients.

**Methods:**

A total of 81 ASO patients (median 20.6 years, IQR 13.5–28.4) underwent CMR for 4D and 2D flow analysis. CMR global longitudinal strain (GLS) feature tracking was compared to echocardiographic (echo) speckle tracking. Agreements between and within tests were expressed as intra-class correlation coefficients (ICC).

**Results:**

Eleven ASO patients (13.6%) showed AR > 5% by 4D flow, with good correlation to 2D flow assessment (ICC = 0.85). 4D flow stroke volume of the aortic valve demonstrated good agreement to 2D stroke volume over the mitral valve (internal validation, ICC = 0.85) and multi-slice planimetric LV stroke volume (external validation, ICC = 0.95). 2D flow stroke volume showed slightly less, though still good agreement with 4D flow (ICC = 0.78) and planimetric LV stroke volume (ICC = 0.87). GLS by CMR was normal (− 18.8 ± 4.4%) and demonstrated good agreement with GLS and segmental analysis by echocardiographic speckle tracking (GLS = − 17.3 ± 3.1%, ICC of 0.80).

**Conclusions:**

Aortic 4D flow and CMR feature tracking GLS analysis demonstrate good to excellent agreement with 2D flow assessment and echocardiographic speckle tracking, respectively, and can therefore reliably be used for an integrated and comprehensive CMR analysis of aortic valve competence and LV deformation analysis in ASO patients.

## Introduction

The arterial switch operation (ASO) with the Lecompte technique is the first-choice surgical technique for correction of transposition of the great arteries (TGA) [1, 2]. Despite overall good clinical outcome, aortic regurgitation (AR) is a common finding and a minority of patients requires aortic valve (AV) and/or aortic root reconstruction [3–5]. Also, subclinical cardiac dysfunction expressed by reduced global longitudinal strain (GLS) has been described, which may precede future systolic dysfunction [6, 7]. Therefore, strict follow-up of ASO patients continuous to be warranted for aortic and ventricular function.

The changed geometrics of the outflow tracts after ASO [8] make it difficult to visualise all aspects of the heart and to quantify degree of AR with routine echocardiography. Therefore, additional advanced cardiac imaging is recommended by the latest guidelines [5, 9].

Cardiovascular magnetic resonance imaging (CMR) has developed rapidly over the past decades. Newly available CMR techniques include whole heart 4-dimensional flow quantification (4D flow) for AV assessment and feature tracking for left ventricular (LV) strain analysis [10, 11]. In theory, transvalvular 4D flow with annular tracking provides more accurate assessment of eccentric regurgitation jets and improved correlation between flow volumes when compared to conventional 2-dimensional CMR measurements because of correction for valvular through-plane motion and flow angulation [12]. In addition, feature tracking by CMR has proven a robust measure of cardiac deformation, providing a valid alternative for echocardiographic speckle tracking [11, 13]. With the recent availability of semi-automatic analysis tools, these measurements become a clinically relevant alternative to echocardiography for ASO patients with a limited acoustic window.

Since eccentric AR jets are often present after ASO, we hypothesised that 4D flow CMR is more accurate than 2D flow assessment to quantify degree of AR in ASO. Furthermore, we hypothesised that CMR feature tracking is a fast and accurate tool to quantify myocardial deformation in this patient cohort. Accordingly, the purpose of this study was to validate 4D flow assessment of the AV and CMR feature tracking for LV strain analysis, with comparison to 2D flow CMR and echocardiographic speckle tracking, respectively, as part of a comprehensive CMR evaluation protocol of ASO patients.

## Methods

A prospective cross-sectional cohort study was performed between August 2011 and January 2015. Patients eligible for inclusion had underwent an ASO in our center and were older than 12 years of age. Patients with pacemaker, claustrophobia or signs of ischemia during exercise test were excluded. Patients were examined by CMR and echocardiography within 1 week. The local medical ethics committee approved this study. Informed consent was obtained from all patients and additional consent by parents if aged under 16 years. Patient characteristics were obtained from the patient charts.

### Cardiovascular magnetic resonance acquisition

Patients were scanned according to a predefined imaging protocol with gadolinium contrast, without anaesthesia or sedation on a 1.5-T CMR system (Ingenia R4.1.2, Philips Healthcare, Best, The Netherlands).

Routine balanced steady-state free precession cine images were acquired in various orientations (short axis multi-plane, 4-chamber and 2-chamber long axis, right and LV outflow tract views in 2 planes) during repeated end-expiratory breath holds using a balanced turbo field echo sequence (voxel size 1.25 × 1.25 × 8 mm, reconstructed temporal resolution 30 frames/ cycle) to provide anatomical information for post-processing. Multi-plane short axis images were also used for planimetric analysis of stroke volume.

4D flow through the AV and mitral valve (MV) was measured with a retrospectively electrocardiography gated, velocity encoded phase-contrast sequence, during free breathing, without correction for breathing movement, without parallel imaging, with a field of view covering the basal volume, voxel size 3.5 × 3.5 × 3.5 mm, temporal resolution 30 frames/ cycle, velocity encoding 1.8 m/s, flip angle 10^o^, TR: 7.3 ms, TE: 3.9 ms [14]. The scan time was 3.42 min at a heart rate of 75 bpm. Significant degree of AR was defined as more than 5% regurgitation fraction [15]. Quantitative 2D flow across the AV was measured with a retrospectively electrocardiography gated, velocity encoded phase-contrast sequence during end-respiratory breath hold, voxel size 2.5 × 2.5 × 8 mm, temporal resolution 20 frames/ cycle, velocity encoding 2.5 ms, TR: 4.7 ms, TE 2.8 ms [16].

### Cardiovascular magnetic resonance analysis

LV planimetric analysis was performed by manual tracing of endocardial contours on short axis images, using Qmass MR Research edition (version 7.4, Medis, Leiden, The Netherlands) to quantify LV end diastolic volume (LVEDV, corrected for body surface area (BSA)), end systolic volume (LVESV, corrected for BSA), mass and ejection fraction (LVEF).

**Fig. 1:**
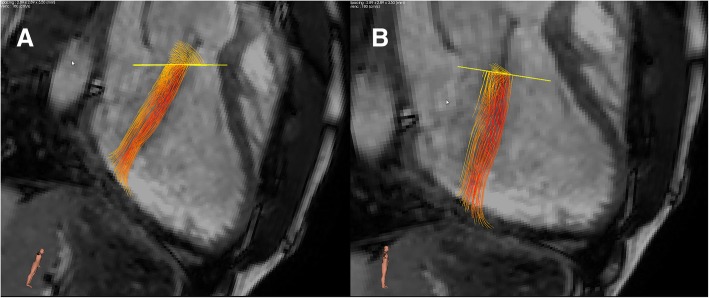
**a** Eccentric aortic regurgitation (AR) with region of interest (ROI) parallel to the valvular plane. **b** Post-processing correction of ROI perpendicular to AR flow direction

4D velocity-encoded CMR data over the AV and MV were analysed using CMR 4D flow (version 2.0, Pie Medical Imaging, Maastricht, The Netherlands) with valve tracking, to quantify flow volume and degree of AR. MV flow assessment was used as internal validation of AV flow volume. For external validation of 4D flow volumes, 2D velocity-encoded CMR data over the AV were analysed using QFlow (version 5.6, Medis). Contours were manually traced in all phases of the 4D and 2D measurements. 4D AR was measured at peak velocity of the jet perpendicular to maximum flow velocity (Fig. 1).

Feature tracking was performed using the 2D Cardiac Performance Analysis MR module of Imaging Arena (version 1.2, TomTec Imaging Systems GmbH, Unterschleissheim, Germany). LV GLS was determined from analysis of the four chamber CMR views, as an average of the six individual segments of the LV [17]. Segments were excluded if non-capturing by the software was observed by eye-balling. If two or more LV segments showed non-capturing, the patient was excluded from analysis.

All CMR analyses were performed by one researcher and subsequently re-analyzed by a second observer to test for inter-observer agreement. Both researchers had 4 years of experience with CMR imaging.

### Echocardiography acquisition and analysis

Doppler transthoracic echocardiography was performed on a Toshiba Artida (Toshiba, Tokyo, Japan) with a 5-MHz transducer. Longitudinal strain analysis was performed with TomTec Image Suite (Version 6.0, TomTec Imaging Systems GmbH). After assessment of full view of all parts of LV myocardium for at least one cardiac cycle, a six-segment analysis was performed. Segments were excluded if eye-balling of the analysis confirmed non-capturing by the software. If two or more segments showed non-capturing, the patient was excluded from analysis. GLS was calculated as an average of the available segments.

All echocardiography analyses were performed by one researcher (4 years of experience), and subsequently re-analyzed by a second observer to test for inter-observer agreement (12 years of experience).

### Statistical analysis

Normality of distribution was checked using the Shapiro Wilk test. Normally distributed continuous data were reported as mean ± standard deviation. Alternatively distributed data were expressed as median (interquartile range). For comparison of groups, independent samples t-test or ANOVA were used as appropriate. For agreement, the difference (between tests and within tests as inter- and intra-observer agreement, respectively) was first checked for significance through one-sample t-test. Agreement was then expressed as two-way, random-effects intra-class correlation (ICC) and checked visually through Bland-Altman plot. ICC was interpreted according to Koo and Li [18]. The coefficient of variance (CoV) was defined as the standard deviation of the differences between the two measurements divided by the mean of these measurements. For all tests a *p*-value of < 0.05 was accepted as statistically significant.

## Results

A total of 81 ASO patients participated in this study. All patients were clinically well and in NYHA class I. Patient characteristics are supplied in Table 1.

**Table 1:** Patient characteristics

Characteristic	Patients (*N* = 81)
Age, years (IQR)	20.6 (13.5–28.4)
Sex, male N (%)	59 (73)
Height, cm	172 ± 12.7
Weight, kg	64.6 ± 17.5
Rashkind, N (%)	56 (69)
Pulmonary banding prior to ASO, N (%)	9 (13)
Age at ASO, days (IQR)	8 (5–18)
Lecompte, N (%)	64 (79%)
Simple transposition, N (%)	58 (72)
Coronary pattern, usual N (%)^a^	67 (83%)
Associated malformations, N (%)	9 (11%)
CoA	3 (4%)
IAA	2 (2%)
TBA	2 (2%)

Data are expressed as mean values ± standard deviation unless stated otherwise

^a^Usual coronary artery pattern defined by Leiden classification (1LCx, 2R)

Abbreviations: *ASO* arterial switch operation, *CoA* coarctation of the aorta, *IAA* interrupted aortic arch, *IQR* interquartile range, *TBA* Taussig Bing anomaly

Systolic LV function was normal as expressed by LVEF as measured by CMR and echocardiography (Table 2). LV dimensions expressed by CMR and echocardiography were also within limits of normal (Tables 2 and 3).

**Table 2:** CMR outcomes

Stroke volume (planimetric), ml	96.8 ± 22
EDV, ml	178.6 ± 42
EDV/BSA, ml/m^2^	101.5 ± 16
ESV, ml	77.7 ± 24
ESV/BSA, ml/m^2^	43.9 ± 10
LVEF, %	58.2 ± 4.7
MV stroke volume (4D), ml	85.9 ± 25
AV stroke volume (4D), ml	92.1 ± 21
AV stroke volume (2D), ml	90.0 ± 21
AV regurgitation (4D), %	4.1 ± 4,1
AV regurgitation (2D), %	3.2 ± 7.1

Data are expressed as mean values ± standard deviation

Abbreviations: *AV* aortic valve, *BSA* body surface area, *EDV* end diastolic volume, *ESV* end systolic volume, *LVEF* left ventricular ejection fraction, *MV* mitral valve

**Table 3:** Strain and echocardiography outcomes

GLS (feature tracking), %	−18.8 ± 4.4
GLS (speckle tracking), %	−17.3 ± 3.1
LVEF, %	55.5 ± 6.1
LVEDD, cm	5.05 ± 0.63
E/E’, %	6.5 ± 1.5
LVmass, g/m^2^	75.4 ± 19.3
Moderate or severe AR, N (%)	5 (6.2)

Data are expressed as mean values ± standard deviation unless stated otherwise

^§^Usual coronary artery pattern defined by Leiden classification (1LCx, 2R)

Abbreviations: *AR* aortic regurgitation, *GLS* global longitudinal strain, *LV* left ventricle, *EF* ejection fraction, *EDD* end diastolic dimension

### CMR flow measurements

4D flow assessment of the AV demonstrated degree of AR of 4.1 ± 4.1% (Table 2), while 11 ASO patients (13.6%) had significant AR exceeding 5%. 2D flow showed comparable regurgitation at 3.2 ± 7.1% (*p* = 0.33), which agreed well with 4D flow (ICC = 0.85, CoV = 9.1%). In patients with significant AR, 4D flow had a tendency for a higher regurgitation fraction than 2D flow (8.6% ± 4.9% vs. 6.3 ± 7.2%, *p* = 0.09). When we dichotomized our ASO patients for 4D flow derived AR below or more than 5%, only indexed LVEDV differed significantly between groups (*p* = 0.014).

**Fig. 2:**
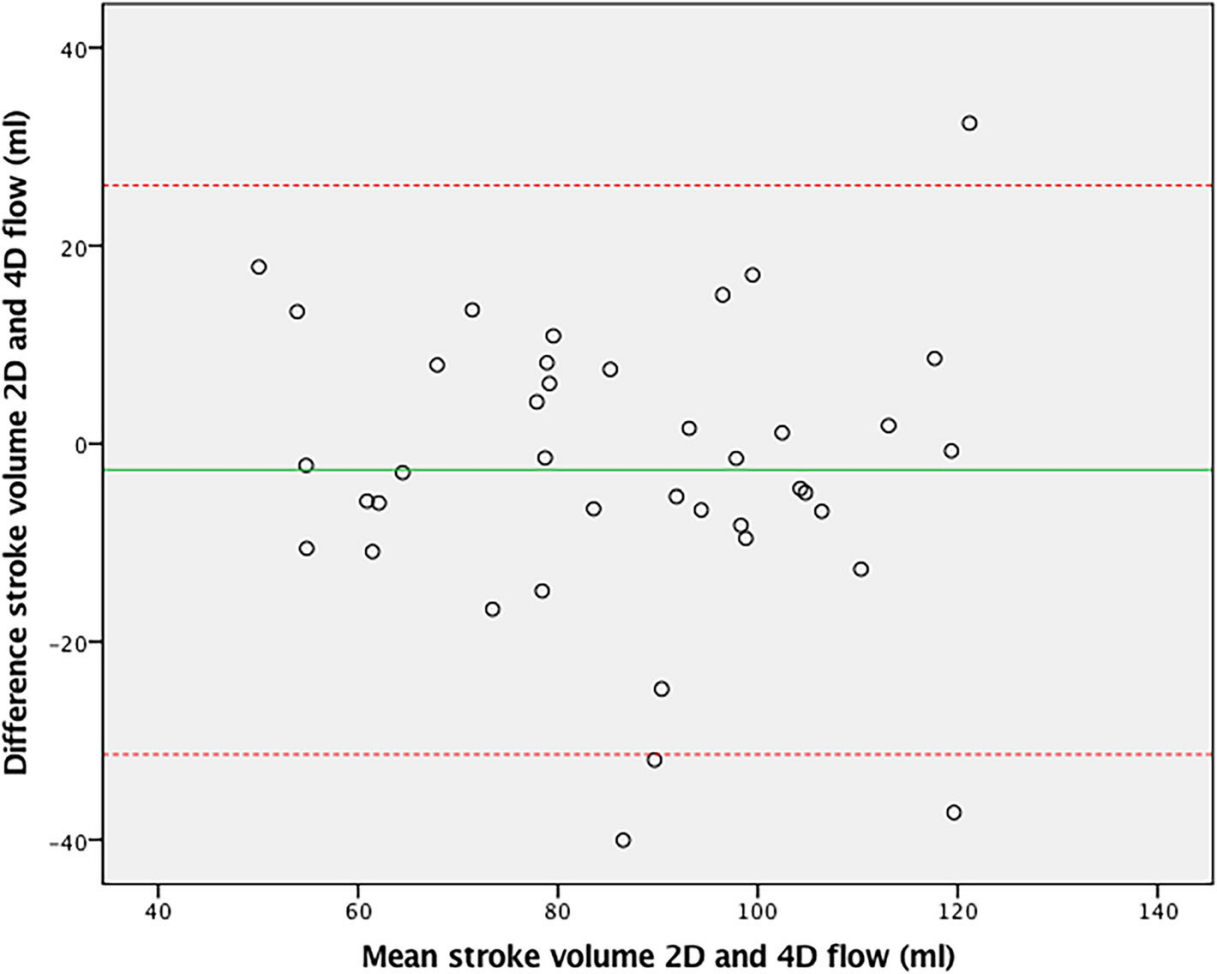
Bland-Altman plot of 4D and 2D flow CMR stroke volumes over the aortic valve

**Fig. 3:**
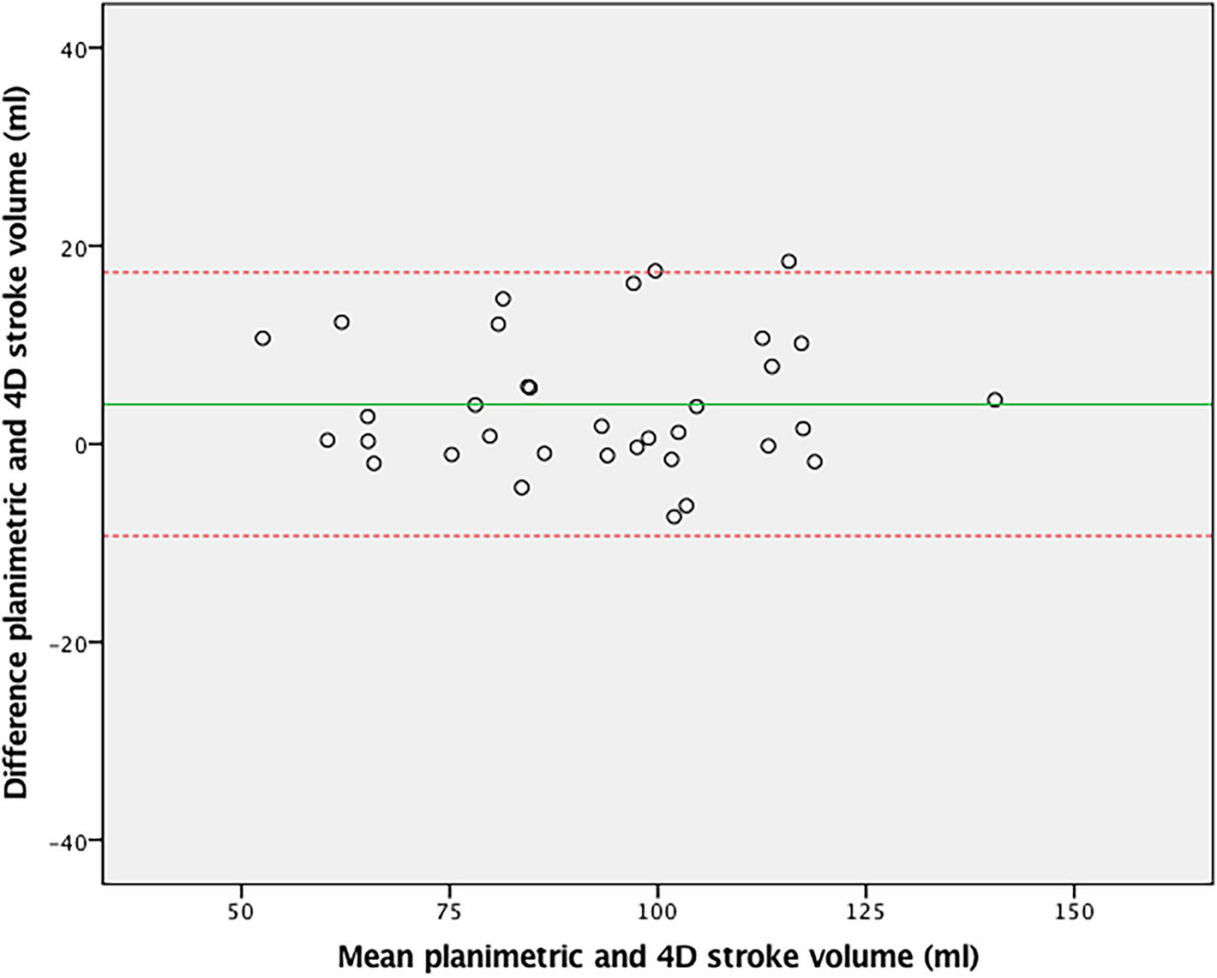
Bland-Altman plot of 4D flow aortic valve and planimetric stroke volume measurements

For external validation of aortic 4D flow assessment, 4D flow derived stroke volume was compared to 2D flow derived stroke volume, demonstrating good agreement (ICC = 0.77, CoV = 11.0%, *p* = 0.20). The Bland-Altman plot is shown in Fig. 2. The outliers in this plot demonstrated good agreement between stroke volumes on 4D aortic, 4D mitral and planimetric measurements (within 5 ml of one another). 2D stroke volume mostly underestimated aortic flow compared to these other methods, despite good imaging quality of the 2D flow measurements in these patients. As a second external validation, 4D flow derived stroke volume was compared to planimetric stroke volume derived from the multi-plane short axis images. Agreement was also excellent (ICC = 0.95, CoV = 6.3%, *p* = 0.26) with the Bland-Altman plot in Fig. 3.

**Fig. 4:**
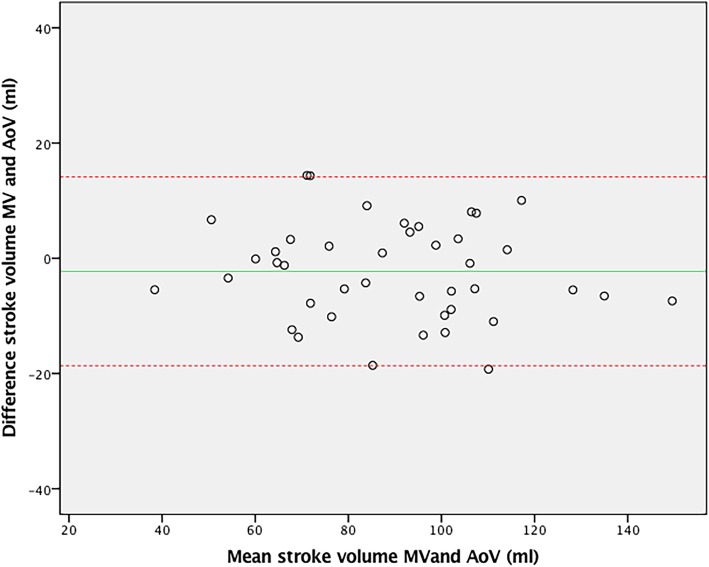
Bland-Altman plot of 4D flow stroke volumes over the mitral valve (MV) and the aortic valve (AoV)

**Fig. 5:**
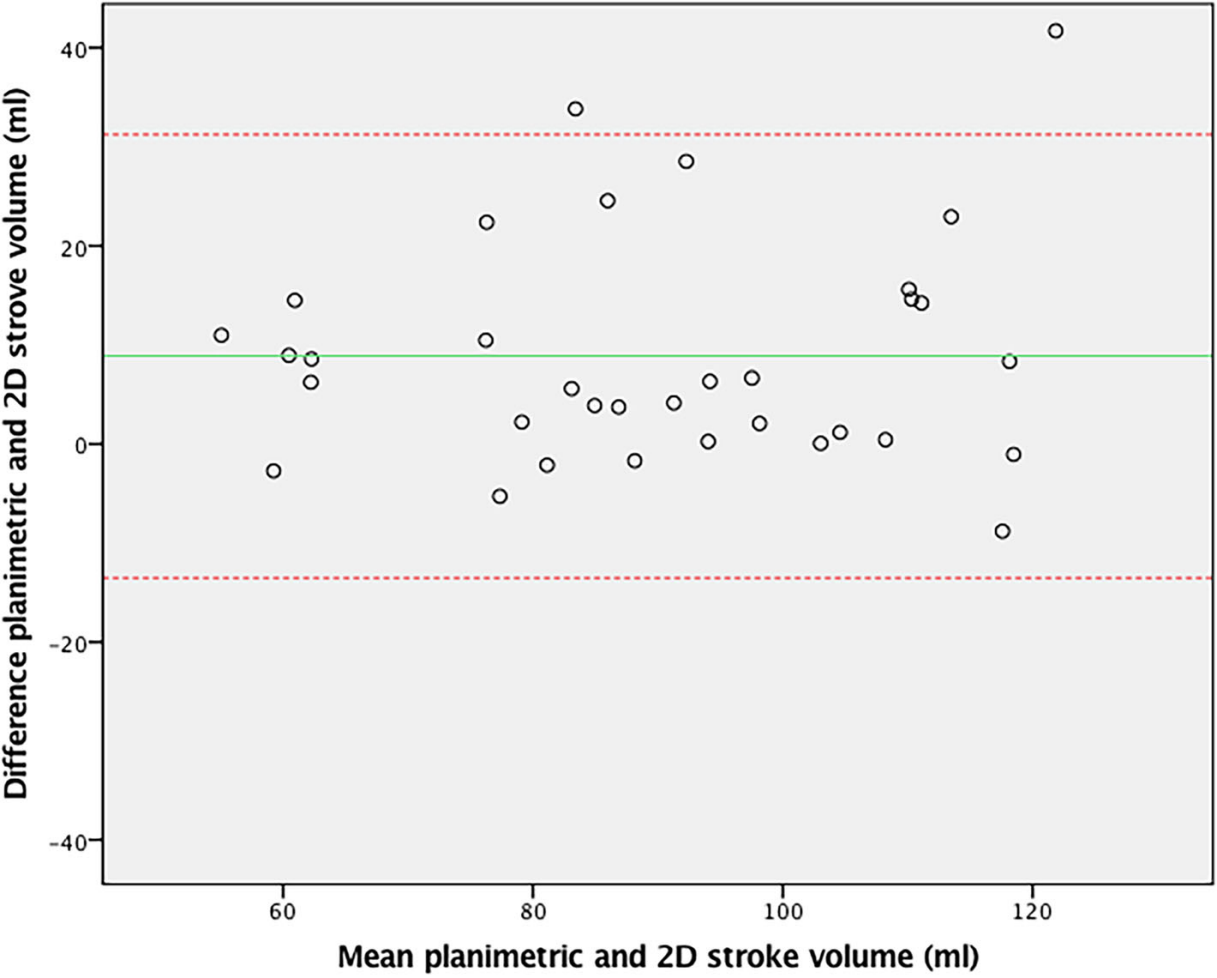
Bland-Altman plot of 2D flow aortic valve and volumetric stroke volume measurements

For internal validation of 4D flow, the 4D stroke volume across the MV (85.9 ± 25 ml) was compared to aortic 4D stroke volume, demonstrating excellent agreement (ICC = 0.94, CoV = 8.2%, *p* = 0.13) (Fig. 4). Inter-observer agreement was excellent for 4D flow stroke volume (ICC = 0.93, CoV = 4.8%, *p* = 0.32), similar to intra-observer agreement (ICC = 0.97, CoV = 2.3%, *p* = 0.58). Compared to 4D flow, the agreement between aortic 2D flow stroke volume and planimetric stroke volume (ICC = 0.87, CoV = 12.7%) was slightly less (*p* = 0.18) (Fig. 5).

### Strain measurements

**Fig. 6:**
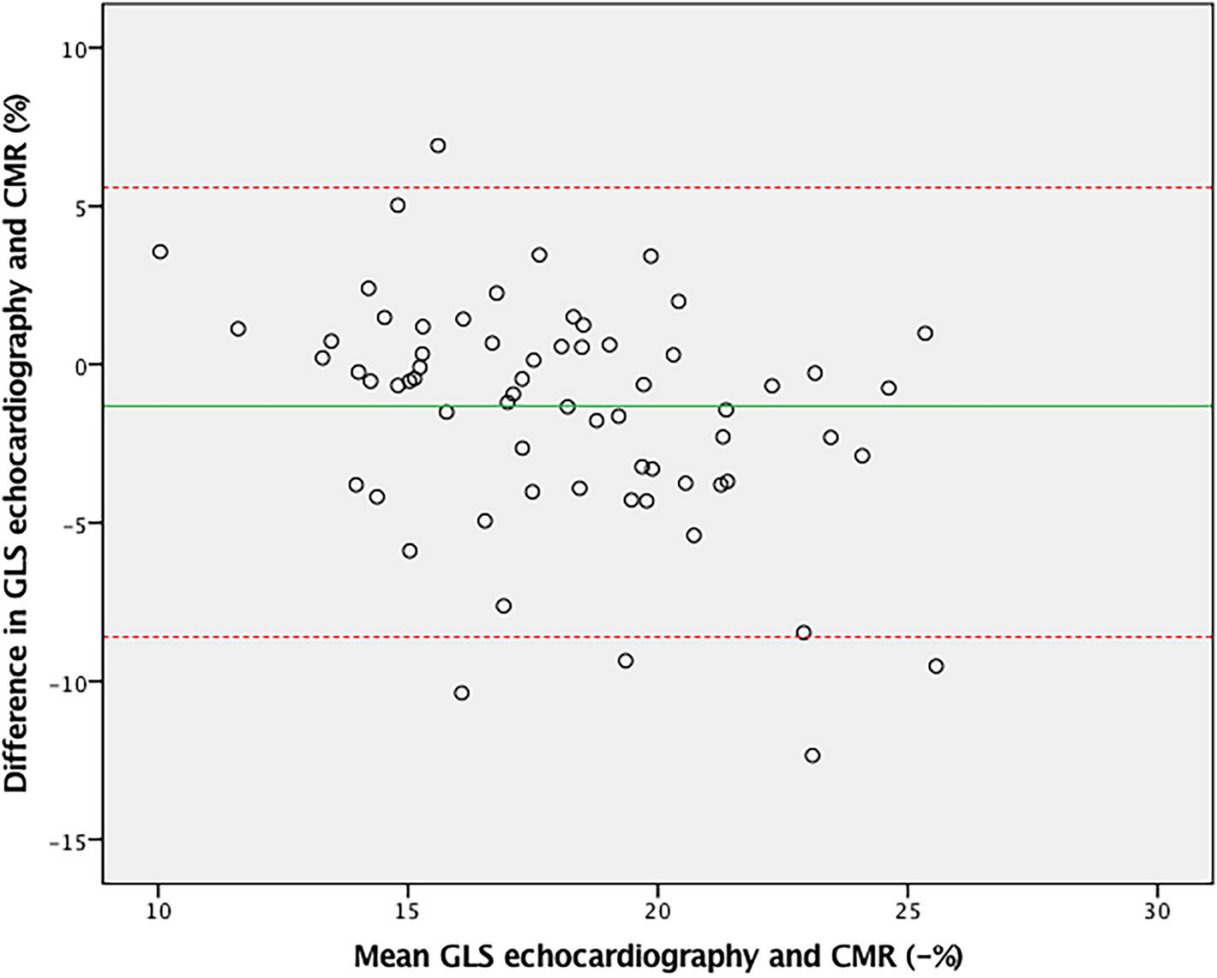
Bland-Altman plot of feature tracking and speckle tracking GLS

To validate GLS by CMR feature tracking, a comparison was made to GLS by echocardiographic speckle tracking (Table 3). Agreement between CMR and echocardiography was good (ICC = 0.80, CoV = 9.6%, *p* = 0.67) as also shown in the Bland-Altman plot (Fig. 6). Inter-observer agreement for GLS assessment by feature tracking was excellent (ICC = 0.94, CoV = 4.2%, *p* = 0.70). Individual LV segmental analysis also showed good agreement between the different segments on feature tracking and speckle tracking (Table 4).

**Table 4:** Segmental GLS values for feature and speckle tracking

Segment	CMR Feature tracking GLS (%), *N* = 75	Echo Speckle tracking GLS (%), *N* = 71
Basal septal	−14.5 ± 3.1	−14.0 ± 3.1
Mid septal	−20.8 ± 3.3	−19.0 ± 3.1
Apical septal	−23.7 ± 3.8	− 22.2 ± 3.9
Apical lateral	−21.3 ± 3.6	−21.0 ± 3.1
Mid lateral	−16.8 ± 3.4	−14.4 ± 3.2
Basal lateral	−16.6 ± 3.2	−15.4 ± 3.0

Data are expressed as mean values ± standard deviation

## Discussion

TGA patients late after ASO frequently demonstrate AR and subclinical cardiac dysfunction, which may precede future systolic dysfunction [3, 4, 6, 7]. Echocardiographic evaluation is often hampered by reduced acoustic window settings and CMR provides a comprehensive alternative to study AV competence and myocardial deformation. The purpose of this study was to validate 4D flow assessment of the AV and feature tracking for LV strain analysis, with comparison to 2D flow CMR and speckle tracking by echocardiography, respectively. In addition, 4D flow potentially allows for earlier detection and more accurate flow assessment. The main findings for this study are:

1. 4D flow is an accurate technique to assess degree of AR and aortic flow volumes in ASO patients, and;
2. CMR feature tracking is a reliable tool to quantify GLS and segmental LS in ASO patients.

### Aortic regurgitation

AR is a well-known complication mid- to long-term after ASO [4]. Although only a minority of ASO patients suffer from significant AR, it may result in reintervention for up to 5% of patients [19]. This AR after ASO is most likely related to aortic root dilation in combination with histological differences in neo-aortic root and valve compositions compared to the native aortic root tissue [20]. Different studies have published conflicting results on the progression rate of AR in ASO patients. However, most recently published reports indicate a slow progression rate of AR over time [3, 19, 21, 22]. Our ASO patients with significant AR demonstrated increased indexed LVEDV when compared to ASO patients with AR less than 5%, as a reflection of increased volume load of the LV.

Agreement between 4D and 2D flow CMR assessment of AR was good in our study. Stroke volume across the AV by 4D flow agreed better with MV flow assessment and planimetric short axis stroke volume measurements than 2D aortic flow, suggesting that 4D flow is a more reliable tool than 2D flow CMR for AV flow assessment. Our findings are in line with Roes et al. [23], who compared 4D flow and 2D flow volumes over the AV and the other intracardiac valves in both healthy volunteers and in patients with AR. 4D flow CMR showed excellent agreement over all intracardiac valves in both groups in their study. 2D flow in healthy subjects demonstrated only average correlation between valves in a study by Kilner et al. [24].

Interestingly, 4D flow gave slightly larger stroke volumes of the LV than 2D flow assessment in our study. In line with this, aortic flow was underestimated by 2D stroke volume in several study participants despite good imaging quality of the 2D flow measurements. In the same patients, all other stroke volume measurements showed good agreement with 4D stroke volume. We were unable to identify common denominators for the cases in which 2D analysis might have underestimated the stroke volume in ASO patients. Jarvis et al. validated 4D flow CMR for quantification of peak velocity across the AV in ASO patients [25]. This study reported higher peak velocities with 4D flow compared to 2D flow CMR, which may explain why 2D flow CMR underestimates flow volumes. This is in agreement with the hypothesis postulated by Roes et al. that eccentric ejection and/or regurgitation jets – which are commonly present in ASO patients – are measured more accurately with 4D flow CMR than with 2D flow CMR [23]. Since 2D flow CMR is measured in a fixed plane, correction for eccentricity of flow and adequate correction for through-plane motion of the aortic root during the cardiac cycle are not possible. With 4D flow CMR the measurement plane is adjusted throughout each phase of the cardiac cycle, as flow is recorded in three orthogonal imaging planes, thereby correcting for both flow eccentricity and through-plane motion of the aortic root [26, 27]. Given the excellent agreement between 4D flow CMR with planimetric flow volumes and the availability of semi-automated analysis of acquisitions with acceptable analysis time, 4D flow CMR appears to be ready for clinical use. Furthermore, despite the significant time expenditure for a 4D flow sequence acquisition (10 to 15 min scan time), the ability to scan both ventricles and great vessels with one sequence provides an important advantage over several individual 2D sequences.

### Global longitudinal strain

Global LV function is generally preserved in ASO patients [4, 28]. Despite this, concerns about long-term preservation of LV function remain, since the myocardial tissue in ASO patients has possibly suffered early damage related to perinatal cyanosis and cardioplegia. Also, altered aortic geometry may increase LV afterload [29]. Impaired deformation expressed by GLS and torsion of the LV has been reported in ASO patients in studies by Pettersen et al. and Di Salvo et al., despite normal LVEF in these studies [6, 7]. GLS has been demonstrated to represent subclinical cardiac dysfunction when abnormal, and is an established precursor of overt ventricular systolic dysfunction [30]. Furthermore, Grotenhuis et al. recently reported diffuse myocardial fibrosis of the LV in ASO patients by use of T1 relaxometry CMR. [Grotenhuis, Eur Heart J 2018] Overt LV failure has not been reported frequently, but follow-up beyond the third decennium after ASO is very limited. Whether the impaired deformation and global myocardial fibrosis will progress into LV failure, remains to be elucidated. Evaluation of systolic LV function should therefore include EF and GLS assessment, as part of long-term follow-up assessment in ASO patients. Although infrequently reported, segmental strain analysis could be used for detection of regional wall motion abnormalities related to prior complications from coronary artery reimplantation [31, 32].

Our study shows good agreement between GLS assessed by CMR and echocardiography, as well as good agreement between the different LV segments, which implies that feature tracking is a valid alternative for speckle tracking. As the acoustic window for echocardiographic analysis can become limited during mid- to long-term follow-up of ASO patients [33, 34] GLS analysis by CMR provides a suitable alternative in this population.

### Limitations

For maximum adherence to clinical practice, the 2D flow CMR data was acquired at 20 frames/ cycle, while 4D and cine images were acquired at 30 frames/ cycle. This may have resulted in lower peak velocity and lower stroke volumes. 2D flow was also acquired on breath hold, where 4D flow was performed with free breathing.

Furthermore, for flow measurements as well as feature tracking, no golden standard was readily available. Therefore, we used consistency in net forward flow volume between consecutive valves as internal reference and multi-slice short-axis planimetric CMR flow volume as best available external reference. Feature tracking on 2D cine images, as used in this study, also has inherent limitations. The temporal resolution is lower than with echocardiography feature tracking and through-plane motion may cause inadequate tracking, both leading to an underestimation of peak deformation [35].

## Conclusion

This study demonstrates that 4D flow CMR is an accurate technique to assess degree of aortic regurgitation and that feature tracking is a reliable tool to quantify GLS in ASO patients. Comprehensive 4D flow and GLS analysis by CMR with semi-automatic analysis tools can therefore reliably be used for an integrated CMR analysis of AV competence and LV deformation analysis in ASO patients in a clinical setting.

## Abbreviations

AR: Aortic regurgitation
ASO: Arterial switch operation
AV: Aortic valve
BSA: Body surface area
CMR: Cardiovascular magnetic resonance
CoV: Coefficient of variance
GLS: Global longitudinal strain
ICC: Intra-class correlation coefficient
LV: Left ventricle/left ventricular
LVEDV: left ventricular end diastolic volume
LVEF: left ventricular ejection fraction
LVESV: left ventricular end systolic volume
MV: Mitral valve
ROI: Region of interst
TGA: Transposition of the great arteries
